# Loss of monomorphic and polymorphic HLA antigens in metastatic breast and colon carcinoma.

**DOI:** 10.1038/bjc.1991.418

**Published:** 1991-11

**Authors:** J. R. Goepel, R. C. Rees, K. Rogers, C. J. Stoddard, W. E. Thomas, L. Shepherd

**Affiliations:** Department of Pathology, University of Sheffield Medical School.

## Abstract

**Images:**


Br. J. Cancer (1991), 64, 880-883 © Macmillan Press Ltd., 1991

### Loss of monomorphic and polymorphic HLA antigens in metastatic breast and colon carcinoma

J.R. Goepel[1,4], R.C. Rees[3,4], K. Rogers[2,4], C.J. Stoddard[2], W.E.G. Thomas[2] & L. Shepherd[3,4]

*Departments of [1]Pathology, [2]Surgery, and [3]Section of Tumour Biology and Immunology, Department of Experimental and Clinical Microbiology, [4]Institute for Cancer Studies, The University of Sheffield Medical School, Beech Hill Road, Sheffield S10 1RX, UK.*

**Summary** MHC class I antigens are intimately involved in intercellular communication, and recognition by cytotoxic T cells. Thus tumour cells that fail to express them may be at a growth or metastatic advantage. A series of ten colorectal and ten breast carcinomas, and their respective lymph node metastases, were examined immunohistologically using monoclonal antibodies (mAb) against both monomorphic and A2 polymorphic determinants, and beta-2-microglobulin (beta 2m). Four colon polypoid adenomas and one liver metastasis were also included in the study. In the colon, all normal tissues and polypoid adenomas stained positively throughout, but 6/10 primary tumours had partial or complete loss of expression of monomorphic determinants using mAb W6/32: two node and the liver metastasis showed less, four more expression. Similar results were seen for beta 2m. HLA-A2 expression was absent or reduced in 4/4 colon tumours and all their metastases. Among the breast tumours, W6/32 staining was absent or reduced in 2/10, and node deposits showed two with less reactivity than their primary. Beta 2m staining was reduced or absent in 8/10 primaries and all the node metastases; in every case in which beta 2m was detected in the primary tumour their corresponding lymph node metastasis showed a decreased expression. HLA-A2 expression was absent or reduced in 3/4 primary breast carcinomas, and all their metastases. These results show that individual human colon and breast carcinomas often have a reduced HLA class I antigen expression, which apparently confers a metastatic advantage.

MHC Class I and II genes encode for proteins which are intimately involved in intercellular communications, and establishing self identity for controlling immunological responses. While MHC class II antigens (in man referred to as HLA-DR, DP or DQ) have limited expression on cells of the immune system, MHC Class I antigens are found on all somatic cells as heterodimeric molecules (Benacerraf, 1981; Linsk & Goodenow, 1986; Schwartz, 1982). The class I MHC molecule consists of three alpha regimes, covalently linked to beta-2-microglobulin (beta 2m), which are encoded by separate genes, and linked prior to expression at the cell membrane (Ploegh *et al.*, 1981; Arce-Gomez, 1978). T cell recognition of antigen at the cell membrane is restricted to cells expressing compatible HLA determinants (Zinkernagel & Doherty, 1979). Similarly, immunological recognition of tumour antigens by cytotoxic T lymphocytes (CTL) is restricted to histocompatible cell types. Failure to express HLA class I, or the expression of inappropriate or altered HLA components on cells, is a possible mechanism whereby tumour cells may escape destruction by antigen specific CTL. Several studies have now shown that many human tumours fail to express antigens recognised by monoclonal antibodies (mAbs) specific for the monomorphic sites of HLA class I molecules (Masucci *et al.*, 1987; Smith *et al.*, 1988; 1989; Broker *et al.*, 1984; Lopez-Nevot *et al.*, 1989; Momburg *et al.*, 1986; Momburg & Koch, 1989; Muller & Stutte, 1988; Natali *et al.*, 1983; Perez *et al.*, 1986). Furthermore, some primary human colon carcinomas fail to express HLA class I polymorphic regions (Rees *et al.*, 1988; Momburg & Koch, 1989); the absence of these haplotypes may also be significant.

Experimental studies in murine tumours have shown that the expression of H2 D and K products is important, and in some ways is linked with metastatic potential. With these immunogenic tumours, those with a high level of MHC antigen expression show low metastatic ability; transfection and subsequent expression of the H2K gene into otherwise highly metastatic H2K-negative tumour cell variants reduces tumour growth, and prevents metastasis (Eisenbach *et al.*, 1983; 1984; 1985). Studies on the expression of human MHC class I antigens in primary *vs* metastatic tumour are limited; for example malignant melanoma, digestive tract, and laryngeal tumours (Ruiz-Cabello *et al.*, 1989; Esteban *et al.*, 1989). In these studies, both increased and decreased expression of monomorphic determinants of HLA class I was observed in the metastases compared with their primaries. In the present study we have used mAbs to evaluate a series of human primary colon and breast carcinomas, and their lymph node metastases, for the expression of monomorphic and polymorphic HLA class I determinants. Normal colon mucosa and, where possible, colon adenomas and liver metastases were included in the study. Many of the primary tumours at both sites showed reduced staining for monomorphic and polymorphic HLA class I determinants; metastases showed both greater and lesser degrees of loss of HLA expression when compared with their primary.

### Materials and methods

### *Patients, tissue typing and specimen collection*

Patients admitted to the study were undergoing surgery for carcinoma of the breast or large bowel as determined on clinical grounds. The series represents a collection of cases made available to the investigators, and is not a consecutive or other unselected series. The nature of the study dictates that only cases with node metastases were acceptable, which means the tumours were relatively advanced, and generally of high grade malignancy. Patients with colorectal carcinoma were HLA typed at A and B loci using a standard microlymphocytotoxicity assay; breast tumours were typed by immunostaining for HLA A2. Tissue samples were obtained from operation specimens for carcinoma of the colon, rectum, or breast. For colorectal specimens samples of tumour, polypoid adenomas and liver metastasis (if present), normal colon 5 cm and 15 cm from the tumour, and lymph node were wrapped in aluminium foil, sprayed with 'Freezit' (Sorrisol, Merseyside) until frozen, and then stored at −80°C. For mastectomies, samples of tumour, normal breast and lymph node were taken and stored in a similar manner. All diagnoses were confirmed by conventional paraffin sections before acceptance into the study.

### *Immunoperoxidase staining of frozen sections*

Cryostat sections were cut at 5–10 microns thickness: spare sections were stored at −80°C until used later. After warm-

Correspondence: R.C. Rees.
Received 19 November 1990; and in revised form 17 June 1991.

ing to room temperature, a standard immunoperoxidase method was used: 40 microlitre of primary antibody in tris buffered saline was applied to the sections, and incubated at 4°C overnight. A positive reaction was detected by the avidin-biotin complex method, with diaminobenzidine as the final chromogen. All sections contained intrinsic positive and negative control areas, and negative controls lacking primary antiserum were also included. The mAbs used as primary antisera are listed in Table I. All sections were interpreted by two observers (J.R.G. and L.S.) and equivocal or failed reactions were repeated. Several patterns of staining were seen: if all the tumour cells showed membrane staining the result was expressed as positive ( + ), and if none did it was negative ( – ) (only plasma membrane staining was considered relevant). Partial staining could be a generally faint reaction (compared with intrinsic control cells), or patchy positive staining either as part of the specimen, or groups of cells; this was recorded semi-quantitatively as +/– if more, or –/+ if less was stained.

**Figure 1** Colon primary tumour; partial loss of MHC class I monomorphic determinants. Positive tumour cells near centre, majority are negative; stroma is positive (Immunoperoxidase mAb W6/32).

### Results

### *MHC class I monomorphic antigen determinants*

A series of ten colonic (or rectal) tumours was obtained. In nine cases tumour was detected in an adjacent lymph node; four polypoid adenomas and one liver metastases were also sampled. Using the mAbs W6/32 (monomorphic backbone determinants), and anti beta 2m, the pattern of MHC class I monomorphic antigen expression was assessed. Staining with the other mAbs (PA2.6 and BBM 1) was generally not so successful: positive reactions were usually weaker than with the corresponding other mAb, and there were more failed and negative reactions; in no instance was there a positive reaction when the other mAb was negative. For these reasons the results of staining with PA2.6 and BBM 1 will not be presented or discussed further.

**Table I** Monoclonal antibodies to HLA class I

| *Antibody* | *Specificity* | *Source* |
|---|---|---|
| W6/32 | HLA, A, B, C, monomorphic | Dr Keith Gelsthorpe Sheffield Blood Transfusion Service (Barnstable *et al.*, 1978) |
| PA 2.6 | HLA, A, B, C monomorphic | Imperial Cancer Research Fund (Brodsky *et al.*, 1979*b*) |
| Anti-β2m | β2m | Sera-Tech Ltd. Mouse mAb (clone-SLR-2) |
| BBM.1 | β2m | Imperial Cancer Research Fund (Brodsky *et al.*, 1979*a*) |
| 151-8.1 | HLA A2/28 | Dr Keith Gelsthorpe Sheffield Blood Transfusion |

All normal tissue stained positively with W6/32 and anti beta 2m mAb, whereas primary tumour was either partly or wholly negative in six out of ten samples stained with W6/32 (Figure 1), and four out of six stained with anti beta 2m. Polypoid adenomas were uniformly positive with W6/32 or anti beta 2m mAb. Two lymph node metastases expressed less HLA reactivity than their primary tumours (Figure 2), while four further lymph node metastases had an increased expression of HLA compared with the primary tumour. Using anti beta 2m mAb two lymph node metastases showed a loss of reactivity compared with their primary tumour, while one metastasis showed an increased expression (Table II).

Table III shows the results of a similar study using primary tumour and lymph node metastases from a series of ten human breast cancers. In these tissues two primary tumours showed a partial or complete loss of reactivity using W6/32 mAb. Two lymph node metastases had reduced staining with W6/32 when compared with their primary. A more pronounced loss of reactivity was observed using anti beta 2m mAb in both primary and lymph node metastases. With the primary breast carcinomas four tumours showed a complete absence of anti beta 2m staining, and a further four out of the ten demonstrated a partial loss of reactivity. The lymph node metastases showed a complete absence of beta 2m in eight out of ten tumours: with the remaining two samples both the primary tumours were uniformly positive, while their metastases showed only partial expression of beta 2m.

### *MHC class I HLA A2 polymorphic antigen determinants*

Eight patients (four colon, four breast carcinomas) were positive for A2, either on tissue typing or by demonstrating

**Table II** HLA class I monomorphic antigen expression in primary and metastatic colonic tumours and polyps

| | | Reactivity with mAb | | | | | | | | | |
|---|---|---|---|---|---|---|---|---|---|---|---|
| | | W6/32 | | | | | Anti-β2m | | | | |
| *Case no.* | *Grade*[a] | *Tumour* | *LN Met* | *Liver Met* | *Polyp* | *n* | *Tumour* | *LN Met* | *Liver Met* | *Polyp* | *n* |
| 1 | M | –[b] | –/+ | | | + | + | +/– | | | + |
| 2 | M | – | –/+ | | + | + | –/+ | – | | + | + |
| 3 | P | –/+ | + | | + | + | – | – | | + | + |
| 4 | P | +/– | + | | | + | NT | + | | | |
| 5 | M | + | –/+ | | | + | NT | NT | | | |
| 6 | – | +/– | +/– | | | + | + | + | | | + |
| 7 | M | +/– | | | + | + | +/– | | | + | + |
| 8 | M | + | + | | | + | + | + | | | + |
| 9 | M-P | + | + | | | + | + | + | | | + |
| 10 | W | + | –/+ | –/+ | + | + | +/– | + | – | + | + |

[a]Tumours graded as poor, moderately or well differentiated. [b]Indicates positive ( + ), complete absence ( – ) or mixed positive and negative ( +/– ) staining patterns. NT = not tested.

a b

**Figure 2** Colon primary **a**, positive membrane staining; lymph node metastasis **b**, negative for MHC class I monomorphic determinants, while stroma (intrinsic control) is positive (Immunoperoxidase mAb W6/32).

**Figure 3** Breast primary tumour; partial loss of MHC class I HLA A2 polymorphic determinant (Immunoperoxidase mAb A2/28).

**Table III** HLA class I monomorphic antigen expression in primary breast carcinomas and their nodal metastases

| Case | Tumour size (mm) | Grade[a] | Reactivity with mAb W6/32 Tumour | W6/32 LN Met. | Anti-β2m Tumour | Anti-β2m LN Met. |
|---|---|---|---|---|---|---|
| 1 | 50 | P | –[b] | – | – | – |
| 2 | 38 | M-P | +/– | – | – | – |
| 3 | 30 | P | + | + | – | – |
| 4 | 28 | P | + | + | – | – |
| 5 | 38 | P | + | + | + | +/– |
| 6 | 28 | M | + | + | + | –/+ |
| 7 | 30 | P | + | +/– | –/+ | – |
| 8 | 30 | M | + | + | –/+ | – |
| 9 | 30 | P | + | + | +/– | – |
| 10 | 16 | M | + | + | –/+ | – |

[a]Tumour graded as poor, moderately, or well differentiated. [b]Indicates positive (+), complete absence (–) or mixed positive and negative (+/–) staining patterns.

**Table IV** Expression of HLA-A2 polymorphic antigen in primary breast and colon carcinoma and metastasis deposits

| Tumour type | Case no. | A2/28 Monoclonal antibody Reactivity Tumour | LN Met. | | | Normal tissue/areas |
|---|---|---|---|---|---|---|
| Breast carcinomas | 7 | +/–[a] | –/+ | | | + |
| | 8 | + | +/– | | | + |
| | 9 | +/– | +/– | | | + |
| | 10 | – | – | | | + |
| | | Tumour | LN Met | Liver Met. | Polyp | Normal |
| Colon carcinomas | 7 | – | NT | | | + |
| | 8 | –/+ | +/– | | | + |
| | 9 | – | – | | + | + |
| | 11 | – | – | – | + | + |

[a]Indicates positive (+), complete absence (–) or mixed positive and negative (+/–) staining patterns. NT = not tested.

uniformly positive normal tissue; all of the colon and three of the breast tumours showed partial (Figure 3) or complete loss of reactivity (Table IV). The lymph nodes from these breast cancer patients generally had less expression of HLA A2 antigen; three showed partial, one complete loss of reactivity. Of the four colon tumour samples, three primary tumours demonstrated a complete absence of HLA A2 antigen, the fourth a partial loss. A similar staining pattern was observed in the lymph node metastases; a liver metastasis was negative, whilst the normal mucosa and a polypoid adenoma were positive.

### Discussion

The loss or aberrant expression of HLA class I monomorphic or polymorphic determinants on tumour cells would theoretically allow them to escape cytotoxic attack by lymphocytes mediating antigen specific recognition. Whilst altered expression has been reported in a wide range of human tumours, studies to assess this in relation to metastasis are few. Using mAbs against monomorphic HLA determinants and beta 2m, examinations of melanoma, squamous carcinoma of larynx, and colorectal adenocarcinoma have shown divergence and loss of expression of class I HLA between primary tumours and their metastases (Esteban *et al.*, 1989; Ruiz-Cabello *et al.*, 1989). There was also correlation between the grade of differentiation and expression amongst the laryngeal carcinomas, with loss in poorly differentiated tumours. In some instances the lack of HLA class I expression is due to a loss of gene activity, as shown in a recent study (Momberg & Koch, 1989) which demonstrated the absence of beta 2m protein from human colon carcinomas to be associated with loss of messenger RNA.

In a previous report we have documented the loss of both monomorphic and polymorphic HLA class I determinants in human colon carcinoma (Rees *et al.*, 1988); in several cases primary tumours that stained with mAb W6/32 failed to stain with a mAb specific for the appropriate haplotype (A2 or Bw4). The presence study evaluates further the expression of HLA class I molecules in primary and metastatic deposits from individual patients with colon or breast carcinoma. Although partial or total loss of reactivity was observed in tissue containing tumour, no consistent pattern could be established between the primary and metastatic tumour in individual patients. Both an increase and a decrease in HLA

expression was seen in colon carcinoma lymph node deposits when compared with their primaries; the one liver metastasis showed a decrease, and the staining of colon polypoid adenomas was identical to normal mucosa. With breast carcinomas, although there was some loss of reactivity in the metastases for HLA class I monomorphic antigen (W6/32 staining), a decrease in staining with anti-beta 2m was seen. Though this was a striking feature of the breast carcinomas, it is probably not a true indication of decreased beta 2m, in view of the non-covalent linkage between it and the rest of the class I MHC molecule identified by W6/32. Immunohistology is at best only semi-quantitative, and the reduced staining may reflect a lower affinity for the antibody. The lack of expression of HLA A2 polymorphic antigen determinants was confirmed in most of the relevant colon carcinomas; while some of the breast metastases showed loss compared with their primaries. Although only eight cases were examined, these observations provide further evidence which suggests that loss of class I antigenic determinants is preferentially associated with metastasis. The cases in this study have been selected because metastases were available; the expression of HLA antigens in the primary tumours has not been compared with its expression in tumours of similar size and grade without known metastases – features known to correlate with clinical outcome (and thus metastasis) in breast carcinoma (Haybittle *et al.*, 1982). In a study by Ruiz-Cabello *et al.* (1989), where class I expression was studied in colon, melanoma and epidermoid tumours, divergence of expression of class I was seen between primary tumours and their autologous metastases. Of the six cases reported, only two metastases showed a decrease in the intensity of staining with W6/32 monoclonal antibody compared with the primary tumour. A further report by this group (Lopez-Nevot *et al.*, 1989) reported an apparent decrease in the number of tumour cells expressing monomorphic HLA antigens in right colon carcinoma metastases.

In colorectal, gastric and laryngeal carcinomas a selective loss of HLA-B isotype determinants occurred in tumours previously classified as HLA positive (Lopez-Nevot *et al.*, 1989). Although differences in HLA expression between tumour types is observed, a consensus of opinion supports the view that both monomorphic and polymorphic HLA determinants are decreased in metastatic tumour deposits compared with its autologous primary tumour. In spite of the relationship between HLA expression and immunity, it may well be that its loss in tumours with an enhanced metastatic phenotype is a reflection of biological events other than those of an immunological nature. For example, they interact with some growth factor receptors and cytoskeletal proteins, and are capable of modulating secondary and tertiary cell signalling pathways (Haliotis *et al.*, 1990); this may confer a metastatic advantage.

We wish to thank the Trustees of Weston Park Hospital and the Yorkshire Cancer Research Campaign for supporting this work.

### References

ARCE-GOMEZ, B., JONES, E.A., BARNSTABLE, C.J., SOLOMON, E. & BODMER, W.F. (1978). The genetic control of HLA-A and B antigens in somatic cell hybrids: requirements for beta-2-microglobulin. *Tissue Antigens*, **ii**, 96.

BARNSTABLE, C.J., BODMER, W.F., BROWN, G. & 4 others (1978). Production of monoclonal antibodies to group A erythrocytes, HLA and other human cell surface antigens: new tools for genetic analysis. *Cell*, **14**, 9.

BENACERRAF, B. (1981). Role of MHC products in immune regulation. *Science*, **212**, 1229.

BROCKER, E.B., SUTER, L. & SORG, C. (1984). HLA-DR antigen expression in primary melanomas of the skin. *J. Invest. Dermatol.*, **82**, 244.

BRODSKY, F.M., BODMER, W.F. & PARHAM, P. (1979). Characterisation of a monoclonal anti-beta2-microglobulin antibody and its use in the genetic and biochemical analysis of major histocompatibility antigens. *Eur. J. Immunol.*, **9**, 536.

BRODSKY, F.M., PARHAM, P., BARNSTABLE, C.J., CRUMPTON, M.J. & BATMER, W.F. (1979*b*). Monoclonal antibodies for analysis of the HLA system. *Immunol. Rev.*, **47**, 3.

EISENBACH, L., SEGAL, S. & FELDMAN, M. (1983). MHC imbalance and metastatic spread in Lewis lung carcinoma clones. *Int. J. Cancer*, **32**, 113.

EISENBACH, L., HOLLANDER, N., GREENFELD, L., YAKOR, H., SEGAL, S. & FELDMAN, M. (1984). The differential expression of H-2K versus H-2D antigens, distinguishing high-metastatic from low-metastatic clones, is correlated with the immunogenic properties of the tumour cells. *Int. J. Cancer*, **34**, 567.

EISENBACH, L., HOLLANDER, N., SEGAL, S. & FELDMAN, F. (1985). The differential expression of class I major histocompatibility complex antigens controls the metastatic properties of tumour cells. *Transplant. Proc.*, **17**, 729.

ESTEBAN, F., CONCHA, A., HUELIN, C. & 4 others (1989). Histocompatibility antigens in primary and metastatic squamous cell carcinoma of the larynx. *Int. J. Cancer*, **43**, 436.

HALIOTIS, T., CARLOW, D.A. & ELLIOTT, B.E. (1990). Nonimmunological aspects of MHC function in the regulation of cell proliferation and the malignant phenotype. *J. Cancer Cells*, **2**, 86.

HAYBITTLE, J.L., BLAMEY, R.W., ELSTON, C.W. & 5 others (1982). A prognostic index in primary breast cancer. *Br. J. Cancer*, **45**, 361.

LINSK, R.L. & GOODENOW, R.S. (1986). Immunological and non-immunologic roles of the major histocompatibility complex in tumorigenesis. *Cancer Rev.*, **6**, 40.

LOPEZ-NEVOT, M.A., ESTEBAN, F., FERRON, A. & 6 others (1989). HLA class I gene expression on human primary tumours and autologous metastases: demonstration of selective losses of HLA antigens on colorectal, gastric and laryngeal carcinomas. *Br. J. Cancer*, **59**, 221.

MASUCCI, M.G., TORSTEINDOTTIR, S., COLOMBANI, J., BRAUTBAR, C., KLEIN, E. & KLEIN, G. (1987). Down-regulation of class I HLA antigens and of the Epstein Barr virus-encoded latent membrane protein in Burkitt lymphoma lines. *Proc. Natl Acad. Sci.*, **84**, 4567.

MOMBURG, F., DEGENER, T., BACCUS, E., MOLDENHAUER, E., HAMMERLING, G.J. & MOLLER, P. (1986). Loss of HLA-A, B, C and de novo expression of HLA-D in colorectal cancer. *Int. J. Cancer*, **37**, 179.

MOMBURG, F. & KOCH, S. (1989). Selective loss of beta-2-microglobulin mRNA in human colon carcinoma. *J. Exp. Med.*, **169**, 309.

MULLER, H. & STUTTE, H.J. (1988). The correlation of major histocompatibility complex gene product expression with proliferative activity and metastatic competence in ductal carcinoma of the breast. *Verh. Dtsch. Ges. Path.*, **72**, 260.

NATALI, P.G., GIACOMINI, P., BIGOTTI, A. & 4 others (1983). Heterogeneity in the expression of HLA and tumor-associated antigens by surgically removed and cultured breast carcinoma cells. *Cancer Res.*, **43**, 660.

PEREZ, M., CABRERA, T., LOPEZ NEVOT, M.A. & 4 others (1986). Heterogeneity of the expression of class I and II HLA antigens in human breast carcinoma. *J. Immunogenet.*, **13**, 247.

PLOEGH, H.L., ORR, H.T. & STOMINGER, J.L. (1981). Major histocompatibility antigens: the human (HLA -A, -B, -C) and Murine (H-2K, H-2D) class I molecules. *Cell*, **24**, 287.

REES, R.C., BUCKLE, A.M., GELSTHORPE, K. & 4 others (1988). Loss of polymorphic A and B locus HLA antigens in colon carcinoma. *Br. J. Cancer*, **57**, 374.

RUIZ-CABELLO, F., LOPEZ NEVOT, M.A., GUTIERREZ, J. & 8 others (1989). Phenotypic expression of histocompatibility antigens in human primary tumours and metastases. *Clin. Expl. Metastasis*, **7**, 213.

SCHWARTZ, R.H. (1982). Functional properties of I region gene products and theories of immune response (Ir) gene function. *Ia Antigens*, Vol 1, 161. Ferrone, S. & David, C.S. (eds), CRC Press: Boca Raton.

SMITH, M.E.F., BODMER, W.F. & BODMER, J.G. (1988). Selective losses of HLA-ABC locus products in colorectal adenocarcinomas. *Lancet*, **i**, 823.

SMITH, M.E.F., MARSH, S.G.E., BODMER, J.G., GELSTHORPE, K. & BODMER, W.F. (1989). Loss of HLA-ABC allele products and lymphocyte function-associated antigen 3 in colorectal neoplasia. *Proc. Natl Acad. Sci. USA*, **86**, 5557.

ZINKERNAGEL, R.M. & DOHERTY, P.C. (1979). MHC-restricted cytotoxic T-cells: studies of the biological role of polymorphic major transplantation antigens determining T-cell restriction-specificity, function and responsiveness. *Adv. Immunol.*, **27**, 51.


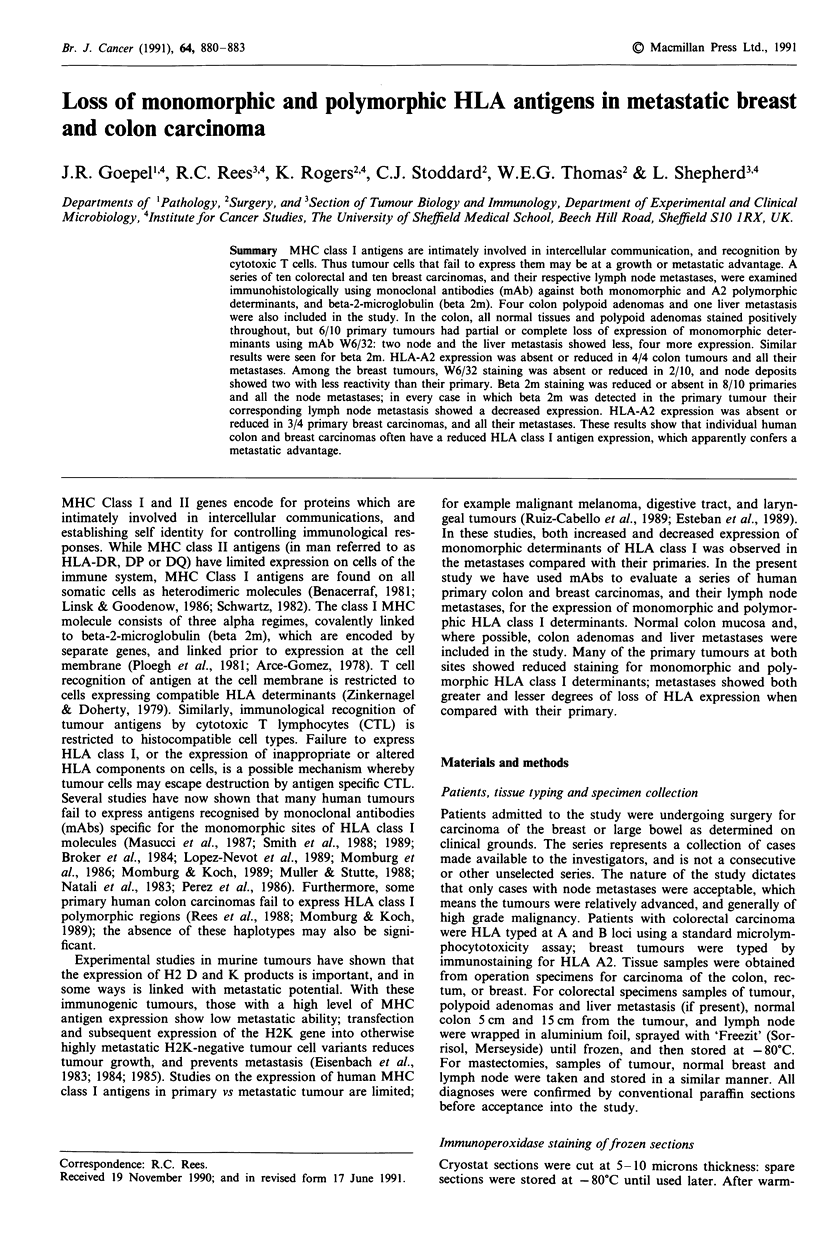

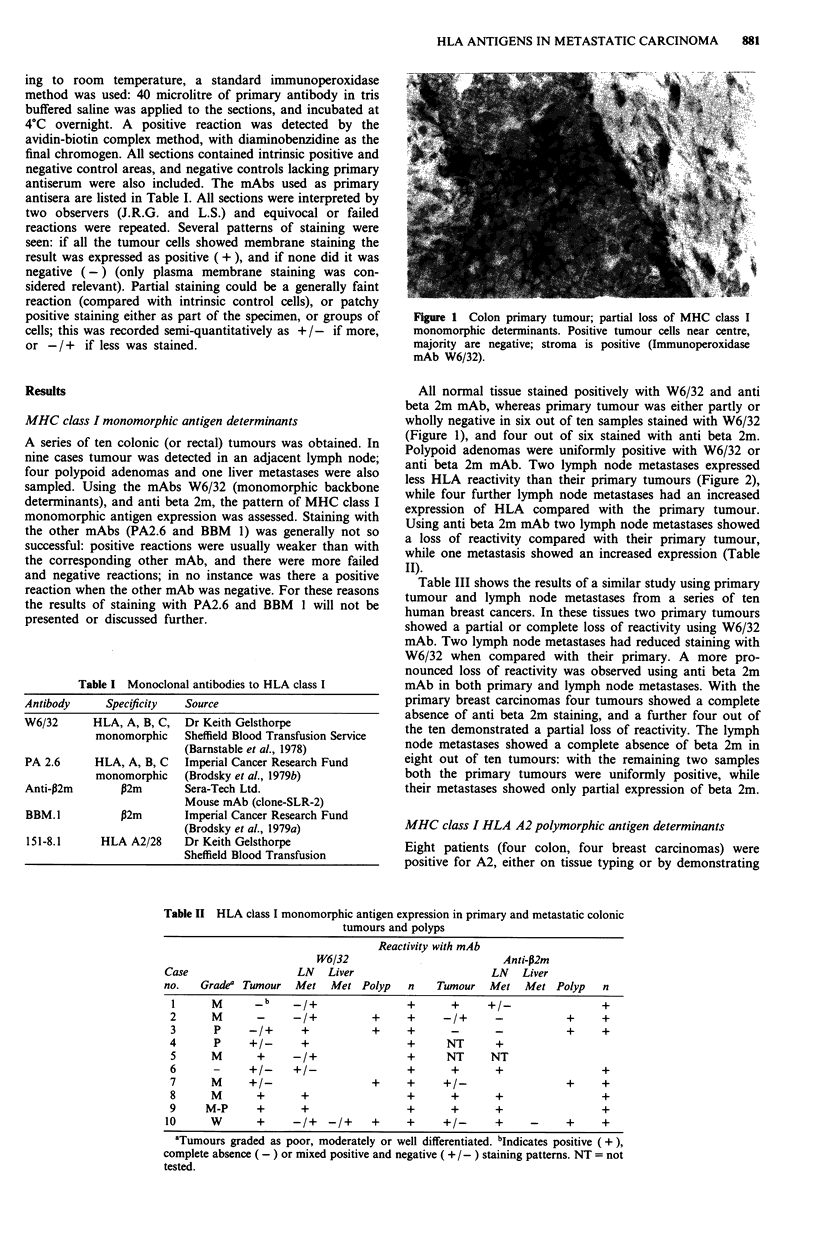

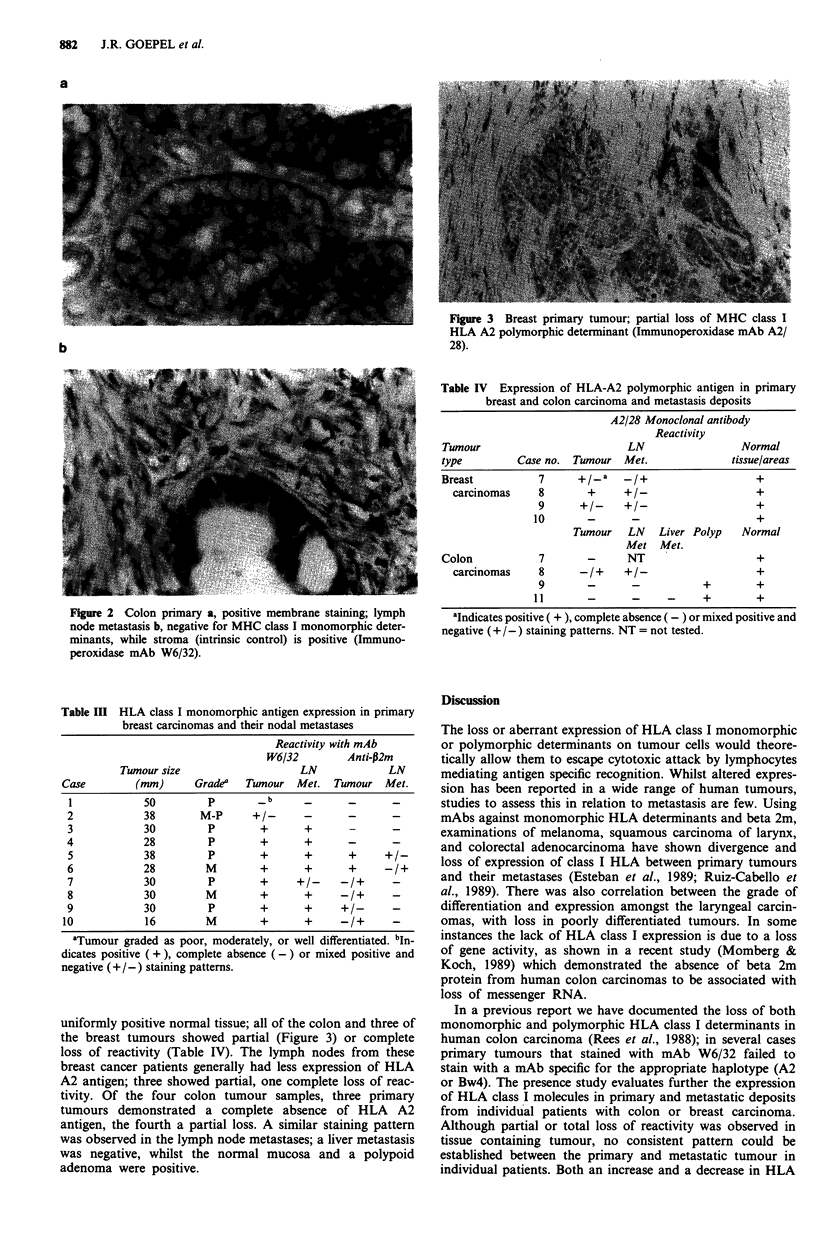

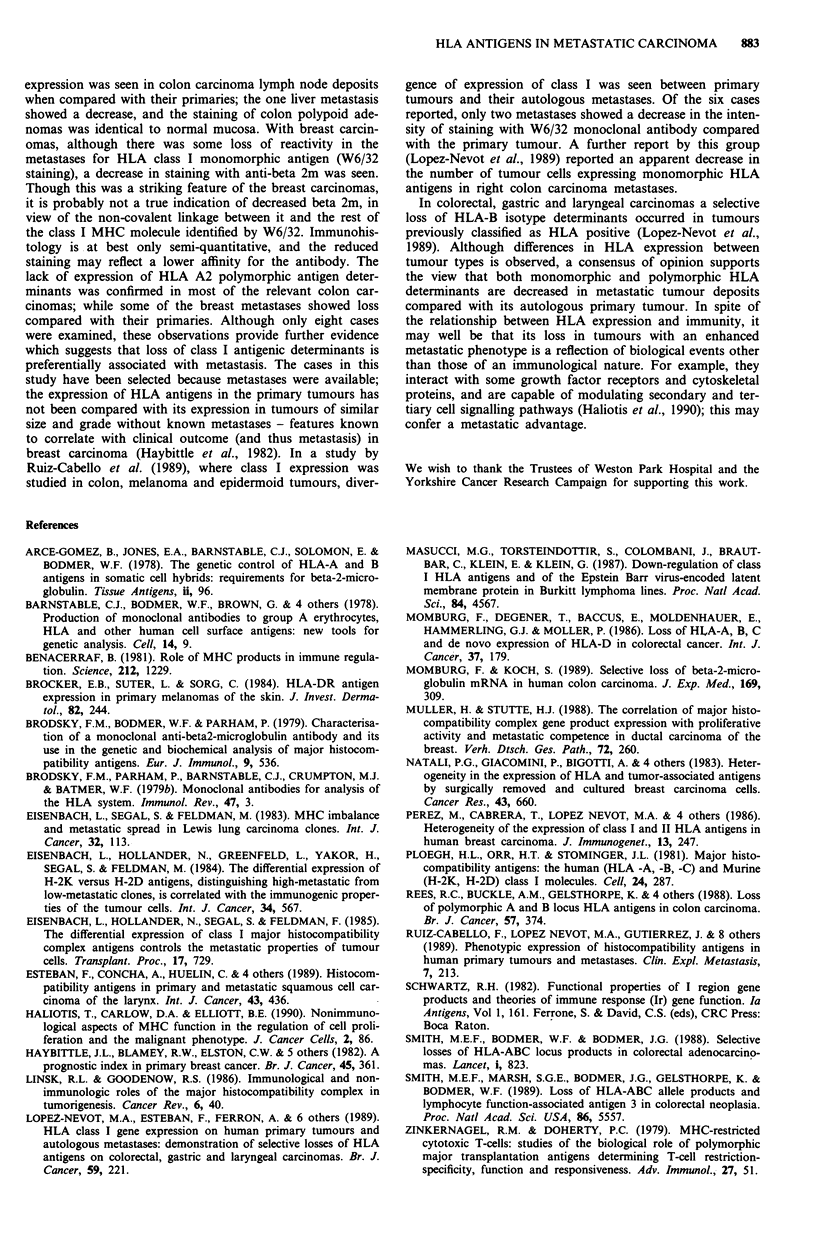


## References

[OCR_00444] Arce-Gomez B., Jones E. A., Barnstable C. J., Solomon E., Bodmer W. F. (1978). The genetic control of HLA-A and B antigens in somatic cell hybrids: requirement for beta2 microglobulin.. Tissue Antigens.

[OCR_00456] Benacerraf B. (1981). Role of MHC gene products in immune regulation.. Science.

[OCR_00465] Brodsky F. M., Bodmer W. F., Parham P. (1979). Characterization of a monoclonal anti-beta 2-microglobulin antibody and its use in the genetic and biochemical analysis of major histocompatibility antigens.. Eur J Immunol.

[OCR_00471] Brodsky F. M., Parham P., Barnstable C. J., Crumpton M. J., Bodmer W. F. (1979). Monoclonal antibodies for analysis of the HLA system.. Immunol Rev.

[OCR_00460] Bröcker E. B., Suter L., Sorg C. (1984). HLA-DR antigen expression in primary melanomas of the skin.. J Invest Dermatol.

[OCR_00481] Eisenbach L., Hollander N., Greenfeld L., Yakor H., Segal S., Feldman M. (1984). The differential expression of H-2K versus H-2D antigens, distinguishing high-metastatic from low-metastatic clones, is correlated with the immunogenic properties of the tumor cells.. Int J Cancer.

[OCR_00476] Eisenbach L., Segal S., Feldman M. (1983). MHC imbalance and metastatic spread in Lewis lung carcinoma clones.. Int J Cancer.

[OCR_00494] Esteban F., Concha A., Huelin C., Pérez-Ayala M., Pedrinaci S., Ruiz-Cabello F., Garrido F. (1989). Histocompatibility antigens in primary and metastatic squamous cell carcinoma of the larynx.. Int J Cancer.

[OCR_00499] Haliotis T., Carlow D. A., Elliott B. E. (1990). Nonimmunological aspects of MHC function in the regulation of cell proliferation and the malignant phenotype.. Cancer Cells.

[OCR_00504] Haybittle J. L., Blamey R. W., Elston C. W., Johnson J., Doyle P. J., Campbell F. C., Nicholson R. I., Griffiths K. (1982). A prognostic index in primary breast cancer.. Br J Cancer.

[OCR_00512] López-Nevot M. A., Esteban F., Ferrón A., Gutiérrez J., Oliva M. R., Romero C., Huelin C., Ruiz-Cabello F., Garrido F. (1989). HLA class I gene expression on human primary tumours and autologous metastases: demonstration of selective losses of HLA antigens on colorectal, gastric and laryngeal carcinomas.. Br J Cancer.

[OCR_00521] Masucci M. G., Torsteindottir S., Colombani J., Brautbar C., Klein E., Klein G. (1987). Down-regulation of class I HLA antigens and of the Epstein-Barr virus-encoded latent membrane protein in Burkitt lymphoma lines.. Proc Natl Acad Sci U S A.

[OCR_00526] Momburg F., Degener T., Bacchus E., Moldenhauer G., Hämmerling G. J., Möller P. (1986). Loss of HLA-A,B,C and de novo expression of HLA-D in colorectal cancer.. Int J Cancer.

[OCR_00532] Momburg F., Koch S. (1989). Selective loss of beta 2-microglobulin mRNA in human colon carcinoma.. J Exp Med.

[OCR_00537] Müller H., Stutte H. J. (1988). MHC-Antigenexpression, Proliferationsaktivität und nodaler Metastasierungsstatus in 184 invasiven duktalen Mammakarzinomen.. Verh Dtsch Ges Pathol.

[OCR_00543] Natali P. G., Giacomini P., Bigotti A., Imai K., Nicotra M. R., Ng A. K., Ferrone S. (1983). Heterogeneity in the expression of HLA and tumor-associated antigens by surgically removed and cultured breast carcinoma cells.. Cancer Res.

[OCR_00554] Ploegh H. L., Orr H. T., Strominger J. L. (1981). Major histocompatibility antigens: the human (HLA-A, -B, -C) and murine (H-2K, H-2D) class I molecules.. Cell.

[OCR_00549] Pérez M., Cabrera T., Lopez Nevot M. A., Gomez M., Peran F., Ruiz-Cabello F., Garrido F. (1986). Heterogeneity of the expression of class I and II HLA antigens in human breast carcinoma.. J Immunogenet.

[OCR_00561] Rees R. C., Buckle A. M., Gelsthorpe K., James V., Potter C. W., Rogers K., Jacob G. (1988). Loss of polymorphic A and B locus HLA antigens in colon carcinoma.. Br J Cancer.

[OCR_00564] Ruiz-Cabello F., Lopez Nevot M. A., Gutierrez J., Oliva M. R., Romero C., Ferron A., Esteban F., Huelin C., Piris M. A., Rivas C. (1989). Phenotypic expression of histocompatibility antigens in human primary tumours and metastases.. Clin Exp Metastasis.

[OCR_00576] Smith M. E., Bodmer W. F., Bodmer J. G. (1988). Selective loss of HLA-A,B,C locus products in colorectal adenocarcinoma.. Lancet.

[OCR_00581] Smith M. E., Marsh S. G., Bodmer J. G., Gelsthorpe K., Bodmer W. F. (1989). Loss of HLA-A,B,C allele products and lymphocyte function-associated antigen 3 in colorectal neoplasia.. Proc Natl Acad Sci U S A.

[OCR_00587] Zinkernagel R. M., Doherty P. C. (1979). MHC-restricted cytotoxic T cells: studies on the biological role of polymorphic major transplantation antigens determining T-cell restriction-specificity, function, and responsiveness.. Adv Immunol.

